# The more you play, the better you feel: a dose–response analysis of pickleball and mental wellbeing in U.S. adults

**DOI:** 10.3389/fpsyg.2025.1676695

**Published:** 2025-10-01

**Authors:** Oluwatoyosi B. A. Owoeye, Joseph Grese, Madeline Stenersen, Ted Yemm, Chris Sebelski, Katie Sniffen

**Affiliations:** ^1^Translational Injury Prevention Lab, Department of Physical Therapy and Athletic Training, Saint Louis University, St. Louis, MO, United States; ^2^Department of Psychology, Saint Louis University, St. Louis, MO, United States; ^3^PEAK Sport and Spine, St. Louis, MO, United States

**Keywords:** health promotion, recreational sports, older adults, mental health, quality of life

## Abstract

**Introduction:**

Participation in sports, including pickleball, has been linked to improved mental health in prior research. However, the potential dose-response relationship between the amount of pickleball played and mental wellbeing has not been examined in the existing literature. We examined if a higher participation level is associated with greater mental wellbeing in pickleball players. In addition, we assessed if the relationship between pickleball participation and mental wellbeing differs by sex, injury history, and age.

**Methods:**

An online survey including questions regarding participation frequency and duration (over the past 12 months), and mental wellbeing using the WHO-5 Wellbeing Index (higher scores indicating greater wellbeing) was administered to 1,667 pickleball players (mean age: 62.8±12.8 years) across the United States. Descriptive statistics and both unadjusted and adjusted linear regression analyses were conducted, controlling for age, sex, injury history, and participation in other sports.

**Results:**

Higher pickleball participation frequency, characterized by ≥ 3 times weekly (vs. ≤ 2 times weekly), was significantly associated with higher mental wellbeing scores (b = 3.3, 95% CI: 1.74.9, p < 0.001) and higher participation duration, characterized by > 2 h per session (vs. ≤ 2 h per session), was significantly associated with higher mental wellbeing scores (b = 3.0, 95% CI: 1.54.4, p < 0.001). Sex did not moderate the association between pickleball participation and mental wellbeing. However, players with a recent injury reported substantially lower wellbeing scores compared to those without an injury history. Age moderated the relationship between participation and mental wellbeing with increasing age boosting mental wellbeing.

**Conclusion:**

This study identified a positive dose-response relationship between pickleball participation and mental wellbeing, with greater frequency and duration of play associated with higher wellbeing scores. The effect was suppressed in players with injury history, but consistent across sexes and strongest among older adults, particularly those aged 63–77. These findings support the potential of pickleball as a low-barrier strategy to promote mental health in aging populations.

## Background

Wellbeing is a multidimensional construct that encompasses psychological, emotional, and social aspects of health that extend beyond the presence or absence of physical illness (Dodge et al., 2012). Wellbeing as a whole has been a central focus of contemporary health research and is now widely recognized as a crucial predictor of positive health outcomes, longevity, and resilience to stressors and trauma (Diener et al., 2017). Public health frameworks now recognize wellbeing as central, a paradigm shift from merely treating illness to fostering positive holistic health across physical, psychological, and social domains (World Health Organization, 2022). Consequently, researchers have begun to explore various determinants of wellbeing, with physical activity emerging as a consistent supporting factor influencing wellbeing across the lifespan. Physical activity is associated with many physical and mental benefits. Increased physical activity has been linked with higher levels of wellbeing across all age groups (Marquez et al., 2020).

Pickleball is a racket sport that combines elements of tennis, badminton, and table tennis. It is played on a court the same size as a badminton doubles court (6.10 m × 13.41 m) with a low net and a plastic perforated ball. Players use a solid paddle that is larger than a ping-pong paddle but smaller than a tennis racket. Given its smaller court, slower ball, and easier learning curve, pickleball has become especially popular across all ages, from youth to older adults. It is currently one of the fastest-growing sports in the U.S. and globally.

The National Department of Health recommends that adults and older adults should get 150–300 min of moderate exercise or 75–150 min of vigorous activity weekly (Piercy et al., 2018). Findings from recent studies support pickleball as a viable form of moderate-intensity physical activity for older adults. Casper et al. found that regular participation in pickleball was associated with increased physical activity, helping older adults meet recommended guidelines for moderate-intensity exercise (Casper and Bocarro, 2023). Additionally, participants reported high levels of enjoyment and social engagement, suggesting pickleball may be a sustainable and appealing physical activity option to promote active aging (Casper and Bocarro, 2023). Similarly, Webber et al. found that both singles and doubles formats of pickleball met moderate-intensity thresholds, highlighting its potential as an accessible and enjoyable way for older adults to achieve recommended activity levels (Webber et al., 2023).

Older adults often face significant psychological challenges, including increased risk of depression, loneliness, and diminished sense of purpose, all of which can negatively impact overall wellbeing (Carpenter and Gatz, 2022). A recent systematic review and meta-analysis highlights that engaging in organized sport is associated with improved quality of life, social connectedness, and reduced depressive symptoms among middle-aged and older adults (Sivaramakrishnan et al., 2024). Participation in sports in general, and pickleball in particular, has been linked to improved mental health in prior research (Cerezuela and Lirola, 2023). A study by Chen et al. (2021) found that just being involved in pickleball was not enough to have an effect on wellbeing; suggesting the possibility of a dose-response effect of pickleball on wellbeing. In tennis, it was found that playing at least weekly was related to decreased moderate-to-severe psychological distress, but playing less was not significantly different than not playing at all (Yu et al., 2024). The potential dose–response relationship between the amount of pickleball participation and mental wellbeing remains unclear, as this has not yet been explored in the existing literature. Moreover, it is unknown whether individual characteristics moderate the relationship between pickleball participation and mental health outcomes.

The objective of this study was to evaluate the dose-response relationship between pickleball and mental wellbeing. Specifically, we examined if a higher participation level (i.e., higher frequency and duration of play), is associated with greater mental wellbeing in pickleball players. A second objective was to consider participant characteristics and if the relationship between pickleball participation and mental wellbeing is influenced by age, sex, injury history, and participation in other sports.

## Methods

### Study design and participants

This cross-sectional study, involving 1,758 players, was a part of the Surveillance in Pickleball players to reduce INjury burden (SPIN) project (Owoeye et al., 2025). This specific study was designed to evaluate the relationship between pickleball participation levels and mental wellbeing. A nationwide survey was administered using QualtricsXM (Provo, Utah), a secure online platform, to collect injury-related data from individuals actively participating in pickleball across the United States. Eligibility criteria required respondents to be United States residents, at least 18 years old, and to play pickleball a minimum of once per month. Individuals who did not meet these requirements were excluded from the study.

To ensure a broad representation, recruitment targeted players of various ages and skill levels. Primary outreach occurred through digital channels, including social media platforms, podcast promotions, local news coverage, and pickleball-focused email newsletters. Additional recruitment efforts involved distributing flyers with QR codes linking to the survey at pickleball clubs, tournaments, university campuses, and community fitness centers. Before accessing the anonymous survey, participants reviewed a recruitment message outlining the study's goals and procedures. Ethical approval for the study was granted by the Saint Louis University Institutional Review Board (IRB #33859).

### Survey instrument and procedures

An online survey was conducted to better understand the effects of playing time and frequency on mental wellbeing. Specifically, the survey collected information regarding demographic variables, play frequency and play duration over the past 12 months, and self-reported mental wellbeing using the WHO-5 Wellbeing Index questionnaire (World Health Organization, 2024). To capture play frequency and duration, players were asked (with multiple choice responses provided): “How often do you play pickleball, on average?” and “What is the average time spent playing pickleball each time you play?” The WHO-5 Wellbeing Index consists of five positively worded items that measure subjective mental wellbeing over the past 2 weeks (e.g., “I have felt cheerful and in good spirits”). Responses are recorded on a Six-point Likert scale ranging from 0 (“At no time”) to 5 (“All of the time”), with total scores ranging from 0 to 25. Individual player responses were converted into percentages prior to data analysis. Higher scores indicate better wellbeing. The WHO-5 has demonstrated good internal consistency, reliability, and construct validity across diverse populations and is widely used in both clinical and research settings (Topp et al., 2015).

### Statistical analysis

Demographic data and independent variables of interest were presented using descriptive statistics of frequency, percentages, mean and standard deviations (SDs), or confidence intervals (CIs). An independent *t*-test statistics was used to access mean differences in mental wellbeing based on pickleball participation levels. Linear regression analyses (unadjusted and adjusted with beta co-efficient and 95% CIs) were conducted to examine the association between pickleball participation levels and mental wellbeing. Adjustments in multivariable linear regression models included age (continuous data), sex, injury history, and participation in other sports as covariates. Model assumptions, including linearity and normality of residuals, were assessed through diagnostic plots. To assess the moderation effect of age in the relationship between a higher pickleball participation level and mental wellbeing, age was categorized into bins of 15 years to visualize trends and to allow for age classification based on age operational groups e.g., young adults (18–32 years) vs. middle-aged (33–62 years) vs. older adults (≥63 years). Statistical significance was set at *p* < 0.05, and all analyses were performed using STATA version 16.1 (StataCorp; College Station, TX, USA).

## Results

A total of 1,667 players (mean age: 62.8 ± 12.8 years [range: 18–89 years], 54.9% female) had complete data for analysis. Participant characteristics are presented in Table 1.

**Table 1:** Participant characteristics.

**Characteristic**	***n* (%)**
**Sex**	
Female	916 (54.9)
Male	751 (45.1)
**Age category (years)**	
18–32	82 (4.9)
33–37	101 (6.0)
48–62	403 (24.2)
63–77	1,000 (60.0)
≥78	81 (4.9)
**Frequency of play**	
≤ 2 times weekly	531 (31.9)
≥3 times weekly	1,136 (68.2)
**Duration of play**	
≤ 2 h per session	865 (51.9)
>2 h per session	802 (48.1)
**Playing experience**	
< 5 years	1,205 (72.3)
≥5 years	462 (27.7)

Table 2 presents the relationship between pickleball participation levels and mental wellbeing. Players who engaged in pickleball more frequently (≥3 times per week) reported significantly higher (*p* < 0.001) mental wellbeing scores (Mean = 77.5) compared to those who played up to twice weekly (Mean = 73.5). Similarly, players with longer play durations (>2 h per session) had significantly higher (*p* < 0.001) wellbeing scores (Mean = 77.7) compared to shorter sessions (Mean = 74.9).

**Table 2:** Pickleball participation levels and mental wellbeing in players.

**Characteristic**	**Mental wellbeing index mean (95% CI)**	**Mental wellbeing index mean difference (95%CI)**	***p*-value**
**Frequency of play**			
≤ 2 times weekly	73.5 (72.1–74.8)	4.0 (2.4–5.6)	< 0.001
≥3 times weekly	77.5 (76.6–78.4)		
**Duration of play**			
≤ 2 h per session	74.9 (73.8–75.9)	2.8 (1.3–4.3)	< 0.001
>2 h per session	77.7 (76.6–78.7)		

Multivariable regression model #1 revealed that players who engaged in pickleball three or more times per week reported significantly higher mental wellbeing scores compared to those who played two or fewer times per week (Table 3). This association remained significant in both unadjusted (*b* = 4.0, 95% CI: 2.4–5.6, *p* < 0.001) and adjusted models (*b* = 3.3, 95% CI: 1.7–4.9, *p* < 0.001). The model also showed significant negative associations for injury history (*b* = −2.8, 95% CI: −4.4 to −1.2, *p* < 0.001) and regular participation in other sports (*b* = −1.6, 95% CI: −3.1 to −0.02, *p* = 0.047). Age was positively associated with mental wellbeing, with an estimated increase of 0.2 points per year (*b* = 0.2, 95% CI: 0.1–0.2, *p* < 0.001).

**Table 3:** Multivariable linear regression analyses showing the association between pickleball play and mental wellbeing.

**Exposure variable**	**Mental wellbeing index before or on top “b”**	***P*-value**
**Multivariable Model 1 with weekly play frequency as primary exposure**	
Weekly play frequency (≥3x vs. ≤ 2x)	3.3 (1.7–4.9)	< 0.001
Injury history (yes vs. no)	−2.8 (−4.4 to −1.2)	< 0.001
Age	0.2 (0.1–0.2)	< 0.001
Regular participation in other sports (yes vs. no)	−1.6 (−3.1 to −0.02)	0.047
**Multivariable Model 2 with weekly play duration as primary exposure**	
Session duration (>2 vs. ≤ 2 h)	3.0 (1.5–4.4)	< 0.001
Injury history (yes vs. no)	−2.7 (−4.3 to −1.2)	0.001
Age	0.2 (0.1–0.2)	< 0.001
Regular participation in other sports (yes vs. no)	−1.5 (−3.1 to 0.1)	0.059

Values are coefficient and 95% CI; for the categorical predictors, coefficients represent mean difference of mental wellbeing scores between the categories of the exposure variables. Positive values indicate better mental wellbeing outcomes while negative values indicate poorer outcomes. For the numeric predictor (i.e., age), coefficients represent the rate at which outcomes change per unit of mental wellbeing score.

Multivariable regression model #2 revealed that players who played for more than 2 h per session reported significantly higher mental wellbeing scores compared to those playing for 2 h or less (Table 3). This association was evident in both unadjusted (*b* = 2.8, 95% CI: 1.3–4.3, *p* < 0.001) and adjusted models (*b* = 3.0, 95% CI: 1.5–4.4, *p* < 0.001). Injury history showed a significant negative predictor of mental wellbeing (*b* = −2.7, 95% CI: −4.3 to −1.2, *p* = 0.001), while age showed a positive association (*b* = 0.2, 95% CI: 0.1–0.2, *p* < 0.001). Participation in other sports showed a borderline significant negative association (*b* = −1.5, 95% CI: −3.1 to 0.1, *p* = 0.059).

Sex did not moderate the relationship between pickleball participation and mental wellbeing (Table 4). The mean mental wellbeing scores and CIs were similar for male and female players with higher pickleball participation frequency and duration. However, players with a previous injury (12-month history) reported significantly (based on CIs not overlapping) lower mental wellbeing scores compared with players with no previous injury history (Table 5).

**Table 4:** The moderation effect of sex in the relationship between higher pickleball participation level and mental wellbeing.

**Exposure variable**	**Mental wellbeing index mean**	**Confidence interval**
**Mental wellbeing scores of players participating 3x or more**		
**weekly**		
Male	77.6	76.3–78.9
Female	77.4	76.2–78.5
**Mental wellbeing scores of players with play duration more**		
**than 2 h per session**		
Male	76.8	75.2–78.4
Female	78.5	77.1–79.9

**Table 5:** The moderation effect of injury history in the relationship between higher pickleball participation level and mental wellbeing.

**Exposure variable**	**Mental wellbeing index mean**	**Confidence interval**
**Mental wellbeing scores of players participating 3x or more**		
**weekly**		
Previous injury	76.3	75.2–77.4
No previous injury	80.2	78.7–81.6
**Mental wellbeing scores of players with play duration more**		
**than 2 h per session**		
Previous injury	76.5	75.2–77.8
No previous injury	80.5	78.7–82.3

**Figure 1:**
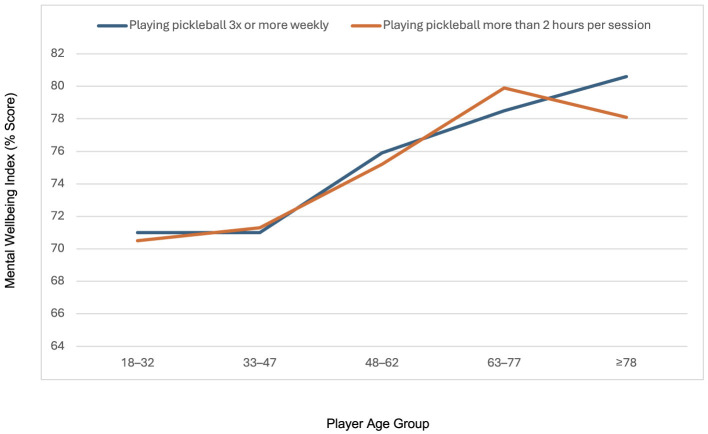
The moderation effect of age in the relationship between higher pickleball participation level and mental wellbeing. A percentage score of zero represents worst possible mental wellbeing and a score of 100 represents best possible mental wellbeing.

Figure 1 demonstrates the moderating effect of age on the relationship between pickleball participation levels and mental wellbeing. Mental wellbeing scores were higher in older age groups, with both frequent play (≥3 times per week) and longer play duration (>2 h per session) being associated with higher wellbeing scores across all age groups. The trend suggests the relationship between pickleball participation and mental wellbeing is stronger among older age groups, peaking in the 63–67 age group before slightly declining in the oldest age category (≥78).

## Discussion

This study examined whether playing more pickleball is linked to better mental wellbeing. Our findings indicate that playing pickleball more frequently and for longer durations is significantly associated with higher levels of mental wellbeing, even after accounting for key covariates such as injury history, age, sex and engagement in other sports. This aligns with existing literature demonstrating the mental health benefits of regular physical activity and recreational sport participation (Eime et al., 2013; Biddle, 2011).

A negative association was observed between injury history and mental wellbeing. A negative association between mental wellbeing and injury history among pickleball players suggests that individuals with a history of injury may experience lower levels of mental wellbeing. This aligns with broader literature across sports contexts showing that musculoskeletal injuries can lead to declines in psychological health due to factors such as pain, loss of function, reduced physical activity, social isolation, and fear of reinjury (Brewer, 2017). In particular, injury in recreational athletes, including those in emerging sports like pickleball, can disrupt not only physical routines but also the social and psychological benefits of sport participation, especially in older adults for whom sport engagement plays a role in cognitive stimulation and emotional support (Rebar et al., 2015). The negative impact of injury on the physical and mental wellbeing of players demands the urgent need for evidence-based injury prevention interventions to be developed and disseminated among players for them to remain active pickleball participants.

Studies have shown that older populations are at a greater risk of decline in wellbeing as they age (Steptoe and Deaton, 2015). In this study, we found a positive association between increasing age and mental wellbeing. This result corroborates previous research showing that older adults who remain physically active tend to experience better psychological outcomes and cope better with the decline in wellbeing that comes with aging (Steptoe and Deaton, 2015; Netuveli, 2008). Our findings emphasize the potential of pickleball not only as a physically engaging activity but also as a meaningful contributor to mental wellbeing, particularly when played consistently. Regular participation in pickleball has been associated with notable psychological benefits for middle-aged and older adults, including enhanced wellbeing, greater life satisfaction, happiness, and reduced depressive symptoms, which are often mediated through increased social integration (Sivaramakrishnan et al., 2024). These positive mental health outcomes are especially valuable given the heightened risk of loneliness, depression, and cognitive decline in this population. As an accessible, low-impact, and socially engaging activity, pickleball offers a meaningful and enjoyable pathway to support mental health and successful aging.

Our analysis suggests that sex does not significantly moderate the relationship between pickleball participation and mental wellbeing. Regardless of frequency or session duration, males and females reported comparable wellbeing scores, with differences that were small and statistically non-significant. This aligns with prior research indicating that the mental health benefits of physical activity are generally consistent across sexes (Chodzko-Zajko et al., 2009). While some studies have reported sex-based variations in exercise motivations and psychosocial outcomes (Molanorouzi and Khoo, 2015), our findings suggest that the mental health advantages of pickleball are equitably experienced by both men and women. These results reinforce the value of promoting pickleball participation broadly, without the need for sex-specific interventions designed to improve psychological wellbeing.

In this study, we found that age moderates the relationship between pickleball participation and mental wellbeing. While higher participation—defined as playing at least three times per week or for more than 2 h per session—was associated with greater mental wellbeing across all age groups, this relationship was most pronounced among older adults, particularly those aged 63 to 67 years. These findings align with prior research indicating that older adults derive significant psychosocial and emotional benefits from regular physical activity and socially engaging recreational pursuits (Everard et al., 2000). The interaction effect pattern observed indicates that while increased participation is associated with increased mental wellbeing, the effect may vary across different life stages. The slight decline observed in the oldest age group (≥78 years) may reflect age-related changes in physical capacity, comorbidities, or social contexts that can moderate the psychological gains of sport participation (Steptoe and Deaton, 2015). Overall, this trend supports the value of promoting age-appropriate, accessible physical activities like pickleball as a strategy to enhance mental wellbeing, especially during later stages of life. Being able to maintain pickleball play as a person age is imperative to maintain wellbeing during the aging process. To support this, injury prevention strategies for the aging population are necessary for continued safe sport participation across the lifespan.

To contextualize our pickleball-related findings, evidence from other recreational sports shows similar associations between participation dose and mental wellbeing. Among older adults, real-world walking-group programs demonstrate a clear dose–response: more sessions attended predict greater improvements in mental and physical health, paralleling our observed gradient with pickleball exposure (Su et al., 2025). Further, racket-sport data, such as tennis, link participation with enhanced wellbeing and reduced depressive symptoms, reinforcing that social, skill-based court sports can deliver mental health benefits comparable to those observed in pickleball (Spring and Holmes, 2020). Importantly, pickleball may hold a distinct advantage by being more accessible, lower-impact, and often perceived as more enjoyable than other sports, suggesting strong potential as an activity that supports long-term participation and sustained mental health benefits.

### Study limitations

There are limitations to this study. First, the average age of survey participants was 63 years, which is higher than the average age of pickleball players currently being reported in the United States (SFIA, 2024). Although the sample in this study included younger participants, it may not be fully representative of all pickleball players in the United States, potentially limiting generalizability. Future studies should prioritize including younger pickleball players and consider including recruitment strategies that involve local pickleball communities across the United States.

Second, the cross-sectional nature of this study limits the ability to infer causality between pickleball participation and mental wellbeing. While associations were observed, we cannot establish whether playing more pickleball improves mental wellbeing, or whether individuals with better mental wellbeing are more likely to engage in pickleball more frequently. Lastly, all data, including pickleball participation and mental wellbeing, were self-reported, which may introduce recall or social desirability bias. Participants may have over- or under-estimated their frequency and duration of play or their mental health status.

### Conclusion

This study found a positive dose—response relationship between pickleball participation and mental wellbeing benefits, with greater frequency and duration of play linked to higher wellbeing scores. Although suppressed in individuals with injury history, the benefits were consistent across sexes and most pronounced among older adults, especially those aged 63–77. These results highlight pickleball as a promising, accessible activity for supporting mental health, particularly in later life. A negative association between injury history and mental wellbeing among pickleball players highlights the need for evidence-based injury prevention strategies to support sustained physical and psychological health across ages.

## Data Availability

The raw data supporting the conclusions of this article will be made available by the authors, without undue reservation.
